# Dose-dependent effect of antipsychotic drugs on autonomic nervous system activity in schizophrenia

**DOI:** 10.1186/1471-244X-12-199

**Published:** 2012-11-14

**Authors:** Yohko Iwamoto, Chiaki Kawanishi, Ikuko Kishida, Taku Furuno, Mami Fujibayashi, Chie Ishii, Norio Ishii, Toshio Moritani, Masataka Taguri, Yoshio Hirayasu

**Affiliations:** 1 Department of Psychiatry, Yokohama City University School of Medicine, 3-9, Fukuura, Kanazawa-ku, Yokohama, 236-0004, Japan; 2 Fujisawa Hospital, Kanagawa Prefecture, Japan; 3 Graduate School of Human and Environmental Studies, Kyoto University, Kyoto, Japan; 4 The Division of Physical and Health Education, Setsunan University, Osaka, Japan; 5 Department of Biostatistics and Epidemiology, Graduate School of Medicine, Yokohama City University, Yokohama, Japan

**Keywords:** Adverse drug effect, Antipsychotic drug, Autonomic nervous system, Heart rate variability, Schizophrenia

## Abstract

**Background:**

Antipsychotic drugs are considered a trigger factor for autonomic dysregulation, which has been shown to predict potentially fatal arrhythmias in schizophrenia. However, the dose-dependent effect of antipsychotic drugs and other psychotropic drugs on autonomic nervous system (ANS) activity remain unclear. The purpose of this study was to investigate the dose-dependent effect of antipsychotic drugs and other clinical factors on ANS activity in an adequate sample size of patients with schizophrenia.

**Methods:**

A total of 211 Japanese patients with schizophrenia and 44 healthy subjects participated in this study. ANS activity was assessed by means of heart rate variability (HRV) power spectral analysis. Antipsychotic drug treatment and various clinical factors were investigated for each participant. The patient group was categorized into three subgroups according to daily dose of antipsychotic drug, and HRV was compared between groups.

**Results:**

The results showed significantly decreased low-frequency and high-frequency components of HRV in the patient group compared to the control group. The high-dose group showed a significantly lower HRV than the medium-dose group and an even lower HRV than the low-dose group. In addition, a significant association between HRV and antipsychotic drug dose was identified by multiple regression analysis. HRV was not associated with age, sex, body mass index, duration of illness, or daily dose of other psychotropic drugs.

**Conclusion:**

These results suggest that antipsychotic drugs exert a significant dose-dependent effect on the extent of decline in ANS activity, and that optimal antipsychotic medication is required to avoid possible cardiovascular adverse events in patients with schizophrenia.

## Background

Increased mortality in patients with schizophrenia compared to the general population has been consistently reported [1-4]. Previous studies have suggested that antipsychotic medications are associated with significant rates of lethal arrhythmias and instances of sudden death [4-7]. Given that both typical and atypical antipsychotic drugs affect the autonomic nervous system (ANS) via neuroleptic effects on various neurotransmitter receptors, antipsychotic drugs are thought to be a trigger factor for autonomic dysregulation. In addition, evidence now exists for an association between decreased ANS activity and increased risk of sudden cardiac death [8-12]. Therefore, it is considered important that clinicians assess ANS activity in patients with schizophrenia treated with antipsychotic drugs to estimate subclinical signs of adverse effects.

Decreased ANS activity in schizophrenia has been reported. Yet, some researchers have suggested an association between low ANS activity and the effects of severity of schizophrenia instead of the effects of antipsychotic drugs [13-17]. These researchers report lower ANS activity in medication-free patients with schizophrenia and lower ANS activity in patients with schizophrenia with severe psychotic symptoms. Only a few studies have attributed decreased ANS activity to medication [18,19]. Those studies suggest that various antipsychotic drugs, which have wide-ranging receptor affinity profiles, decrease parasympathetic nerve activity. However, in those studies, the sample sizes were relatively small or the authors did not consider the dose-dependent effect of antipsychotic drugs on ANS activity or did not assess the influence of coadministered drugs.

The aim of this study was to investigate the dose-dependent effect of antipsychotic drugs on ANS activity in an adequate sample size. We targeted more than 200 patients with schizophrenia under medication for long periods and investigated various clinical factors, including psychotic symptoms, duration of illness, and other psychotropic drugs, that might affect ANS activity.

## Methods

### Subjects

The study protocol was approved by the Institutional Review Board of Seishinkai Fujisawa Hospital and was performed in accordance with the Declaration of Helsinki. All participants provided written informed consent after receiving detailed information on the study.

The study included 211 Japanese patients with schizophrenia (166 inpatients and 45 outpatients; 82 men and 129 women; mean ± standard deviation [SD] age 53.8 ± 14.6 years. These patients met diagnostic criteria for schizophrenia according to the Diagnostic and Statistical Manual of Mental Disorders [20]. Patients with preexisting diseases known to affect ANS activity were excluded. The mean score on the Global Assessment of Functioning Scale [20] (GAF) was 33.7 (SD: 11.7). All of the patients had been already receiving the standard medication before the measurement of ANS activity. All given psychotropic medications on the measurement date of ANS activity, including antipsychotic, anticholinergic antiparkinsonian, and anxiolytic drugs, were registered, and doses were calculated according to standard equivalent conversions [21] of chlorpromazine, biperiden and diazepam. After the measurement of ANS activity, we categorized the patient group into three subgroups according to daily dose of antipsychotic drug in order to study the dose-dependent effects of antipsychotic and other psychotropic drugs on ANS activity. The daily dose in the low-dose group (63 patients; age 57.0 ± 16.2) was ≤500 mg/day; that in the medium-dose group (59 patients; age 53.0 ± 14.8) was 501 to 1000 mg/day; and that in the high-dose group (89 patients; age 52.1 ± 12.9) was ≥1001 mg/day. All procedures were carried out as a part of the standard care.

Control data were obtained from 44 healthy subjects (14 men and 30 women; mean age 48.4 ± 13.4 years). None of the subjects had a history of physical and/or psychiatric disorder or were taking medication.

### Experimental procedure

All measurements were obtained in the same order between 9:00 AM and 11:00 AM in a quiet, comfortable room with minimal arousal stimuli. First, body composition was measured (DS-320; Tanita, Tokyo, Japan), and after appropriate skin preparation, subjects were fitted with electrocardiography (ECG) electrodes and allowed to rest for at least 20 min before measurements were taken. After resting, the CM5 lead ECG electrode was recorded continuously for 5 min with the subject in a sitting position. During the measurement period, all subjects breathed 15 times per min (0.25 Hz) in synchrony with a metronome to ensure that variation in heart rate fluctuation (<0.15 Hz) was respiratory-associated and not due to another source.

### R-R interval power spectral analysis procedure

We used a computer-assisted, 5-min measurement of resting heart rate variability (HRV) to evaluate ANS activity, according to our previous studies [22,23]. The details of our R-R interval power spectral analysis procedures used have been fully described elsewhere [24]. The analytic technique for the present investigation has been applied in basic physiology and clinical research fields, and its validity and reliability have been confirmed in our early studies [23-25]. In addition, the results confirmed those of previous studies, in which Akselrod et al. Pagani et al. and Pomeranz et al. [26-28] conducted experiments on animals or humans with HRV power spectral analysis [29]. The ECG signal was amplified (BBA-8321; Bio-Tex, Kyoto, Japan), digitized via a 13-bit analog-to-digital converter (Daq AD132; Elan, London, United Kingdom) at a 1024-Hz sampling rate, and stored sequentially on a hard disk for later analysis. The stored ECG signal was differentiated, and the QRS spikes and intervals of the impulses (R-R intervals) were detected. The R-R interval data were aligned to obtain equally spaced samples with an effective sampling frequency of 2 Hz and displayed on a computer screen for visual inspection [30]. The direct current component and trend were then completely eliminated with a digital band-pass filter (0.03–0.4 Hz). After passing the data through a Hamming-type data window, power spectral analysis was performed by means of fast Fourier transform on a consecutive 256-s time series of R-R interval data obtained during the test. The spectral powers in the frequency domain were quantified by integrating the areas under the curve for the following respective bandwidth measurements: low-frequency (LF; 0.03–0.15 Hz) component of HRV representing both sympathetic and parasympathetic nerve activity; high-frequency (HF; 0.15–0.4 Hz) component of HRV associated almost entirely with parasympathetic nerve activity; total power (TP; 0.03–0.4 Hz) representing overall ANS activity; and LF/HF ratio representing relative sympathetic nerve activity [26-28].

### Statistical analysis

All statistical analyses were performed with SPSS for Windows Version 11.5 (SPSS, Chicago, IL). We first assessed group differences in clinical characteristics (age, sex, and body mass index (BMI)) between patients with schizophrenia and control subjects with the Student’s unpaired *t*-test or the chi-squared test, and we compared both the LF and HF components of HRV, and the LF/HF ratio between patients with schizophrenia and control subjects with the Student’s unpaired *t*-test. In addition, we categorized the patient group into three subgroups according to daily dose of antipsychotic drug. To assess the difference of ANS activity between groups, variables were analyzed by analysis of variance (one-way ANOVA), with both the LF and HF components of the HRV as the outcomes. *Post-hoc* comparisons employed the Scheffé’s test. In addition, for patients with schizophrenia, we analyzed the influence of clinical factors on ANS activity by the multiple regression analysis, using both the LF and HF components of the HRV as dependent variables, and age, sex, BMI, GAF score, duration of illness, antipsychotic drug dose (chlorpromazine equivalent: CPZeq [21]), anticholinergic antiparkinsonian drug dose (biperiden equivalent: BPDeq [21]), and anxiolytic drug dose (diazepam equivalent: DZPeq [21]) as independent variables. Owing to skewed data, logarithmic transformation was performed on the absolute units of spectral components of the HRV before statistical analysis. A value of P < 0.05 was considered statistically significant.

## Results

### Comparison of patients with schizophrenia and controls

Demographic and medication data for all subjects are listed in Table 1. There was a statistically significant difference in mean age between the schizophrenia group and the control group (patient: 53.8 ± 14.6; control: 48.4 ± 13.4; *P* = 0.027). There were no differences in sex or mean BMI between the two groups. Given antipsychotic drug dose was 1078.0 ± 882.5 mg/day (CPZeq) for the schizophrenia group. In addition, given anticholinergic antiparkinsonian drug dose and given anxiolytic drug dose was 3.2 ± 1.5 mg/day (BPDeq) and 11.1 ± 7.3 mg/day (DZPeq), respectively. Various antipsychotic drugs were prescribed. The most common was risperidone (47.5%), followed by levomepromazine (37.9%) and chlorpromazine (27.5%). The rate of antipsychotic polypharmacy was 72.0%.

**Table 1:** Demographic and medication data for all subjects

	**Controls (n = 44)**	**Patients (n = 211)**
Female/male	30/14	129/82
Age (years)	48.4 ± 13.4	53.8 ± 14.6
Body mass index	23.1 ± 3.6	22.9 ± 4.3
CPZeq*^1^ (mg)	N/A*^4^	1078.0 ± 882.5
BPDeq*^2^ (mg)	N/A	3.2 ± 1.5
DZPeq*^3^ (mg)	N/A	11.1 ± 7.3

Data are presented as mean ± standard deviation (SD).

^*1^The daily dose of antipsychotic drug was converted to approximate chlorpromazine equivalent.

^*2^The daily dose of anticholinergic antiparkinsonian drug was converted to approximate biperiden equivalent.

^*3^The daily dose of anxiolytic drug was converted to approximate diazepam equivalent.

^*4^Not available.

Electrophysiologic results are presented in Table 2. We confirmed normality of both the LF and HF components of HRV. Results of the Student’s unpaired *t*-test showed that both the LF and HF components of HRV in the patient group were lower than those in control group (LF: *P* < 0.001; HF: *P* < 0.001). There were no differences in the LF/HF ratio between the two groups (*P* = 0.511).

**Table 2:** Comparison of absolute (log) power values of the frequency bands for low frequency (LF), high frequency (HF), and LF/HF between patients and controls

	**lnLF**	**lnHF**	**lnLF/HF**
Control group (n = 44)	4.66 ± 0.94	4.25 ± 1.41	0.41 ± 1.15
Patient group (n = 211)	3.50 ± 1.62	3.23 ± 1.60	0.29 ± 1.12
*P* value	<0.001*^1^	<0.001*^2^	0.511

Data are presented as mean ± standard deviation (SD).

*^1, 2^Significant difference (*P* < 0.05, Student’s *t*-test).

### Effects of antipsychotic medication on ANS activity in patients with schizophrenia

**Figure 1:**
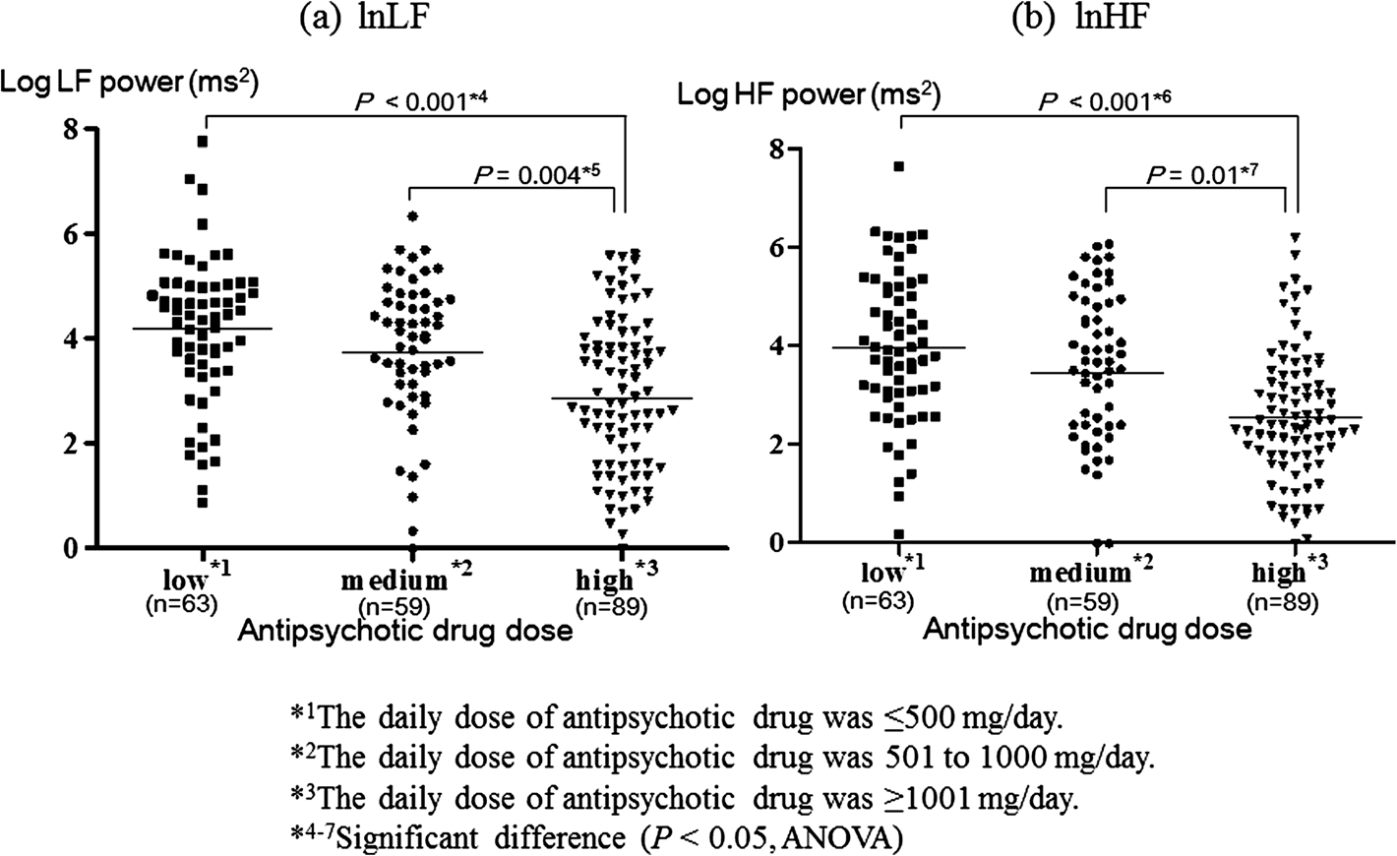
**Comparison of (log) power value of the frequency bands for low frequency (LF) and high frequency (HF) between the three subgroups of patient group.** Lateral bars show mean for each group.

We categorized the patient group into three subgroups according to daily dose of antipsychotic drug (Figure 1). There were no differences in age, sex, mean BMI or duration of illness among three groups except for GAF scores. The high-dose group showed a significantly lower HRV than the medium-dose group (LF: *P* = 0.004; HF: *P* = 0.01) and an even lower HRV than the low-dose group (LF: *P* < 0.001; HF: *P* < 0.001). The medium-dose group showed a significantly lower HRV than the control group (LF: *P* = 0.02; HF: *P* = 0.048), as did the high-dose group (LF: *P* < 0.001; HF: *P* < 0.001).

### Association between ANS activity, psychotropic drug dose, and other factors

The multiple regression analysis for the schizophrenia group showed a statistically significant association between both the LF and HF components of HRV and CPZeq antipsychotic drug dose (LF: *P* = 0.048; HF: *P* = 0.011) (Table 3). There was no significant association between the spectral components of HRV and BPDeq anticholinergic antiparkinsonian drug dose or DZPeq benzodiazepine anxiolytic drug dose. There was also no association between the spectral components of HRV and age, sex, BMI, GAF score, or duration of illness.

**Table 3:** Multiple regression analyses of ANS activity, age, sex, Body mass index, GAF, duration of illness, CPZeq, BPDeq, and DZPeq as independent variables

	**ANS activity**			
	**lnLF**		**lnHF**	
**Independent variable**	**β**	***P***	**β**	***P***
Age	−0.197	0.375	−0.015	0.938
Sex	0.252	0.086	0.237	0.063
Body mass index	−0.024	0.880	0.047	0.733
GAF	−0.004	0.978	0.046	0.724
Duration of illness	−0.017	0.924	−0.157	0.300
CPZeq*^1^	−0.369	0.048*^2^	−0.415	0.011*^3^
BPDeq*^4^	−0.089	0.584	−0.259	0.070
DZPeq*^5^	−0.201	0.219	−0.151	0.284

*^1^The daily dose of antipsychotic drug was converted to approximate chlorpromazine equivalent.

*^2,3^Significant difference (*P* < 0.05).

*^4^The daily dose of anticholinergic antiparkinsonian drug was converted to approximate biperiden equivalent.

*^5^The daily dose of anxiolytic drug was converted to approximate diazepam equivalent.

## Discussion and conclusion

In this study, we found a markedly lower power of both the LF and HF components of HRV, but not a lower LF/HF ratio in patients with schizophrenia compared to control subjects. The LF component of HRV represents both sympathetic and parasympathetic nerve activity, the HF component represents almost entirely parasympathetic nerve activity, and the LF/HF ratio represents relative sympathetic nerve activity [22,31]. Thus, we found decreased parasympathetic nerve activity, but not decreased sympathetic nerve activity, in patients with schizophrenia compared to healthy controls. Based on HRV power spectral analysis, autonomic dysregulation is a consistent finding in schizophrenia, regardless of medication status [13-15,32]. Our present results are concordant with those of previous studies [18,19,22,33].

Some researchers have suggested an association between decreased ANS activity and the severity of schizophrenia. These authors reported parasympathetic hypoactivity in medication-free patients with schizophrenia [13,34,35] and that parasympathetic nerve activity is significantly decreased when the psychotic state is more pronounced [15-17,36]. A few studies have suggested that suppression of parasympathetic nerve activity is associated with disturbances in cortical-subcortical circuits and other central nervous system pathologies in schizophrenia [13,37]. However, patients in those studies were on medication or had been treated with antipsychotic drugs 1 to 4 weeks before ANS activity measurements. Interrupted or readministered antipsychotic drugs could possibly have had more or less of an effect on ANS activity in patients in those studies.

In contrast, a few studies state that medication affects ANS activity in patients with schizophrenia. Studies have reported that some antipsychotic drugs, such as clozapine, with wide-ranging receptor affinity profiles, show imbalance between sympathetic and parasympathetic nerve activity [18,19] and that other antipsychotics drugs, such as haloperidol, which are relatively free of significant effects on neurotransmitter receptors except dopamine receptors, have no significant effect on ANS activity [19,38]. The results of those studies suggest that the effects of antipsychotic drugs on ANS function are derived from anticholinergic and antiadrenergic activity. In the present study, levomepromazine and chlorpromazine, which have potent anticholinergic and antiadrenergic effects, were commonly used, and many of our subjects were treated with multiple antipsychotic drugs.

Considering the results of previous studies and receptor affinity profiles, it is a consensus view that antipsychotic drugs have more or less of an effect on ANS activity. However, there is little evidence to indicate the dosage of antipsychotic drugs necessary to affect ANS activity. We investigated the dose-dependent effect of antipsychotic drugs on ANS activity. Our results suggest the following. If the daily dose of antipsychotic drugs is ≤500 mg/day, ANS activity in patients with schizophrenia is not significantly lower than that in controls. If the daily dose of antipsychotic drugs is 501 to 1000 mg/day, antipsychotic drugs exert a large effect on ANS activity and decrease ANS activity. If the daily dose of antipsychotic drugs is ≥1001 mg/day, antipsychotic drugs exert a very large effect on ANS activity and decrease ANS activity significantly. In short, the effect might be large if the dose of antipsychotic drug is high. There was a statistically significant difference in mean GAF scores among three subgroups. However, the result of the multiple regression analysis showed that there was no association between the spectral components of HRV and GAF score after adjustment for variables such as age, sex, BMI, duration of illness, CPZeq, BPDeq, and DZPeq (Table 3).

Multiple regression analysis of the schizophrenia group showed a statistically significant association between daily dose of antipsychotic drugs and parasympathetic nerve activity. However, there was no correlation between ANS activity and daily dose of anticholinergic antiparkinsonian or anxiolytic drugs. In the present study, the anticholinergic antiparkinsonian drug dose was 3.2 ± 1.5 mg/day (BPDeq). Anticholinergic antiparkinsonian drugs potentially affect ANS activity; however, the effect might be small unless at a high dose. There was also no association between ANS activity and the severity of the disease or duration of illness. In addition, multiple regression analysis showed that ANS activity was not associated with age, although there was a statistically significant difference in mean age between the patient group and the control group.

To summarize our results, we interpreted our findings as follows. 1) Antipsychotic drugs decreased ANS activity in medicated patients with schizophrenia, presumably mediated by the parasympathetic nervous system. 2) If the antipsychotic drug dose was ≥501 mg/day, antipsychotic drugs had a dose-dependent effect on ANS activity and decreased ANS activity significantly, at least, in medicated patients. 3) Anticholinergic antiparkinsonian drugs did not significantly affect ANS activity unless at a high dose. 4) The severity of schizophrenia did not have an effect on ANS activity.

The present study has some potential limitations. First, although the patient sample size was greater than 200, the number of subjects was still relatively small. Second, we did not analyze the influence of each drug separately because most of our subjects were treated with multiple drugs. Third, because there were no medication-free patients, it is unclear whether the pathomechanisms and/or the severity of schizophrenia directly affect ANS activity. Additional investigations are necessary to clarify these issues.

Our present results suggest that antipsychotic drugs exert a dose-dependent effect on ANS activity and decrease ANS activity significantly and that HRV power spectral analysis, as a diagnostic measurement of ANS, may allow for the identification of patients at high risk for sudden cardiac death. Therefore, we suggest that low ANS activity might be a biomarker for adverse effects of antipsychotic drugs rather than severity of schizophrenia. Prospective studies will be aimed at carefully collecting data from at-risk patients to elucidate the association between subclinically decreased ANS activity and the occurrence of serious adverse effects. These investigations will provide clinical evidence for HRV power spectral analysis as a potential monitoring system to protect psychiatric patients from severe adverse events such as fatal ventricular arrhythmias.

## Pre-publication history

The pre-publication history for this paper can be accessed here:

http://www.biomedcentral.com/1471-244X/12/199/prepub
